# Natural Polymers, Their Modifications and Composites with Synthetic Polymers for Tympanic Membrane Regeneration

**DOI:** 10.3390/jfb16100384

**Published:** 2025-10-13

**Authors:** Roman O. Shaikenov, Polina G. Serbun, Jingran Zhang, Hao Wu, Zuobin Wang, Petr P. Snetkov, Svetlana N. Morozkina

**Affiliations:** 1 Institute of Advanced Data Transfer Systems, ITMO University, Kronverkskiy Prospekt, 49, Bldg. A, Saint Petersburg 197101, Russia; polina_serbun@itmo.ru (P.G.S.); ppsnetkov@itmo.ru (P.P.S.); morozkina.svetlana@gmail.com (S.N.M.); 2 Ministry of Education Key Laboratory for Cross-Scale Micro and Nano Manufacturing, Changchun University of Science and Technology, Changchun 130022, China; 3 Medical Institute, Saint Petersburg State University, Universitetskaya Embankment, 7–9, Saint Petersburg 199034, Russia; 4 Progressive Materials and Additive Technologies Center, Kabardino-Balkarian State University Named After H.M. Berbekov, St. Chernyshevsky, 173, Nalchik 360004, Russia

**Keywords:** natural polymers, eardrum, tympanic membrane, silk fibroin, hyaluronic acid, collagen, bacterial cellulose, biocompatible composites

## Abstract

The perforation of the tympanic membrane is a world-wide clinical problem resulting from trauma or infection and requiring effective regeneration methods. In recent years researchers have focused on natural polymers as promising materials for restoring the structure and function of the tympanic membrane. This review summarizes current advances in the use of natural polymers, such as silk fibroin, chitosan, hyaluronic acid, collagen, bacterial cellulose, alginates and others, for the treatment of tympanic membrane perforations. The key properties of these materials are discussed, including their biocompatibility, biodegradability, mechanical strength, and ability to support cell adhesion and proliferation. The review also covers the potential of natural polymers and their combinations in tympanic membrane regeneration and suggests the directions for future research.

## 1. Introduction

The tympanic membrane (TM or eardrum) is a membrane located between the outer and middle ear. The TM plays a key role in the perception of sound vibrations and the protection of the middle ear cavity, as it separates the external auditory canal from the middle ear cavity and transmits sound vibrations from the external auditory canal to the ossicles. The TM can be anatomically divided into three layers: the epidermal outer layer, the fibrous middle layer, and the mucous inner layer, consisting mainly of keratinocytes, fibroblasts, and collagen [1]. The eardrum varies in thickness in different parts, but the greatest thickness does not exceed 150 µm [2]. This makes the eardrum vulnerable to perforation due to mechanical trauma or infection. Chronic otitis media is one of the main causes of eardrum perforation. Every year, 700 million cases of acute otitis and 31 million cases of chronic otitis are recorded worldwide [3]. And by 2050, it is predicted that 10% of the world’s population will have hearing problems [4]. The body is able to recover with a small perforation on its own within a few days, but if the perforation persists for three months, the patient is diagnosed with chronic perforation [5].

Autologous material is currently a popular material for TM restoration. For example, fat grafts are easy and quick to obtain with minimal risk of complications, demonstrate efficacy comparable to temporal fascia, and promote new vessel formation and tissue restoration through the release of angiogenic and growth factors [6,7]. However, fat grafts have a limited source: they are taken from the earlobe or subcutaneous tissue behind the ear, so abdominal fat is used as an alternative source. However, because surgical treatment is costly, causes side effects, and may be uncomfortable for patients, better techniques are being developed [8].

A significant number of studies have been devoted to solving the problem of tympanic membrane perforation and its regeneration [9,10,11]. For example, a silicone film with a layer of Soft Skin Adhesive (SSA) MG7-1010 has been tested on mouse eardrums in vivo [12]. This technology implies that the artificial structure will remain in the ear permanently, which requires additional investigation to verify the durability of such a film. Another approach is to use decellularized eardrums into which bone marrow stem cells have been placed [13]. In this case, the question arises about the source of the eardrums, since in clinical practice, human eardrums will be needed for this. Attempts to use autologous material have also been made. In one of the studies researchers collected 10 mL of blood from patients, isolated platelet-rich fibrin by the centrifugation, and used it to close the chronic perforation of the eardrum. The method showed excellent results, but some patients experienced inflammation and infection after the surgery. In addition, as the authors admit, this method is only suitable for treating small perforations [14]. There is still potential for research in the field of platelet-rich fibrin membrane use [15,16]. In addition, the use of stem cells alone for TM regeneration has been investigated, but the cells require a matrix for protection and to facilitate proliferation [17].

Natural polymers are promising materials for eardrum regeneration. They combine mechanical strength and the ability to control their properties through chemical modifications with biodegradability and tolerance for the immune system [18]. The use of natural polymers in combination with other materials can result in a synergic effect. In this review, we discussed the main studies on the use of natural polymers for TM regeneration carried out in recent years.

## 2. Natural Polymers Used for Tympanic Membrane Regeneration

Table 1 presents the key information about the studies that used natural polymers for tympanic membrane regeneration.

### 2.1. Silk Fibroin

Silk fibroin is a protein obtained from the cocoon of the silkworm *Bombyx mori*. Silk fibroin is widely used in the development of smart materials and medical materials due to its properties: mechanical strength, biocompatibility, and others [58]. In otolaryngology, silk fibroin is one of the most popular materials for treating tympanic membrane perforations. It is often used in in vivo experiments on animals [59].

Jin Kim and others conducted an in vivo experiment to compare the effectiveness of silk fibroin patches and paper patches for the treatment of large perforations of the tympanic membrane. Because of the advantages of silk fibroin and its ability to retain moisture, the tympanic membranes with fibroin patches recovered from perforation 2 times faster than those with paper patches. The authors believe that this promising method is also suitable for humans [19].

It is worth noting the biodegradability of silk fibroin, which guarantees safe metabolism of the implant. It was demonstrated how this occurs in a culture medium with the L929 cell line (mouse fibroblasts) and when fibroin membranes are implanted under the skin of rats. In the case of the cell line, the degradation of the films did not exceed 17% within 60 days. These results were confirmed by an in vivo experiment: after 19 months, the films had degraded by only 35%. Such implant stability is especially important in the first months after the surgery but may become unnecessary later on [20].

**Figure 1:**
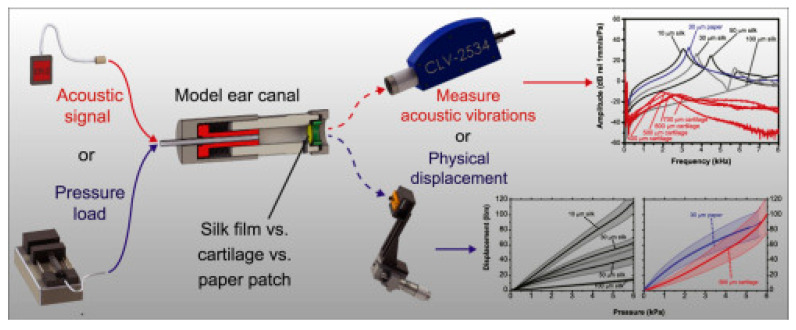
Comparison of the physical and acoustic characteristics of silk fibroin film compared to cartilage and paper patch. Reprinted with permission from Ref. [60] Copyright 2016 Elsevier.

In addition, some studies confirmed that silk fibroin not only promotes the closure of the perforation of the TM but also helps to restore its functions. For example, Benjamin J. Allardyce and colleagues developed an in vitro model of the external auditory canal and middle ear to determine how the material copes with sound transmission and whether it can withstand pressure changes (Figure 1). The silk fibroin membrane performed well on both counts [60].

Yi Shen and colleagues also published two research papers. In the first, they demonstrated that silk fibroin-based scaffolds are significantly more effective than paper patch and the commercial gelatin-based material Gelfoam for TM regeneration [21]. In the second paper, the authors studied how the TM regenerated with different patches transmits sound. It was found that the hearing threshold in animals with fibroin patches returned to the preoperative level, while paper patches led to an increase in the hearing threshold, i.e., the sensitivity of the TM decreased [22].

Some studies have already been conducted on humans, confirming that fibroin patches are more effective in treating chronic TM perforation than standard perichondrial myringoplasty, as the use of fibroin patches is simpler and faster, does not require additional surgical invasions in the patient’s body, better restores sound conduction and promotes natural regeneration of the tympanic membrane. Anyway, further research is still needed to study the treatment of large tympanic membrane perforations on humans [23].

Several attempts have been made to use silk fibroin together with TM cells to create a bioengineered implant [24]. Cell adhesion and cell survival markers showed good results, but the use of cells together with natural polymers has not become one of the leading approaches in the field of TM regeneration.

### 2.2. Chitosan and Chitin

Chitosan is a natural aminopolysaccharide obtained by the deacetylation of chitin. Chitin is a nitrogen-containing polysaccharide that is chemically similar to cellulose. Both polymers are widely used in medicine and chemistry due to their biocompatibility, non-toxicity and other properties, especially chitosan in the field of drug delivery [25].

One simple method that does not require special equipment is the production of chitosan-based films, for example, from aqueous solutions of chitosan and glycerol, which was used as a plasticizer. The solutions were filtered, poured into Petri dishes and dried at 45 °C for 12 h. In an in vivo experiment on rats, the films were attached to the tympanic membrane at the site of a small perforation using an adhesive ointment, and the animals were observed. It was found that the films promote the perforation healing, as 100% of the rats in the experimental group recovered their eardrums within the first week after the experiment. However, in the group without the treatment, spontaneous regeneration within a week was observed in only 78% of the rats. The authors believe that these data will serve as a basis for further work, as it is necessary to find out how the films affect the healing of large perforations [61].

Thus, Hoon Seonwoo and others developed chitosan patches using the aforementioned technology: a solution of chitosan and glycerol was poured into Petri dishes and dried. In addition, the researchers added IGFBP2 to the solution, a protein that binds to insulin-like growth factor and stimulates epithelial progenitor cells, leading to improved proliferation and differentiation of stem cells. IGFBP2 release was 30% over 15 days in vitro. It was also found that patches with IGFBP2 significantly improve cell migration in the wound healing assay. It worth noting that in the in vivo experiment, TM perforations were significant in size: from 10 to 60% of the TM area. It was found that the size of the perforation is one of the key factors in TM regeneration. Perforations of 40% or less showed significant improvement over the 10 weeks of the experiment. Perforations with a larger area did not heal despite the use of patches. Nevertheless, the researchers developed a promising path as implants based on natural polymers: they included growth factors in their composition [28].

Another technology is developing chitosan scaffolds by freezing and lyophilising the polymer solution. The porous polymer mass is then cut into small pieces for further research. An in vitro test showed that the scaffolds support TM cell proliferation. In an in vivo test, TM perforations accounted for 35–50% of the TM area. The researchers compared the effect of the scaffolds with a paper patch. The scaffolds significantly accelerated TM regeneration, which was especially noticeable in the first week of the experiment. Hearing sensitivity was almost completely restored after the treatment with scaffolds, but the researchers did not provide any data on sensitivity in mice treated with paper patches, so it is unknown whether scaffolds are more effective than paper patches. In addition, the thickness of the TM after the treatment with scaffolds was several times thicker than the natural TM and significantly thicker than the TM after the paper patch. This is due to the porous structure of the material and its gradual degradation, which, according to the authors, results in the formation of voids in the TM. Although no bacterial infection was observed, such cavities could become a potential starting ground for pathogens. It is also unknown how such structure affects the mechanical strength of the regenerated TM and its ability to transmit sound vibrations [26].

**Figure 2:**
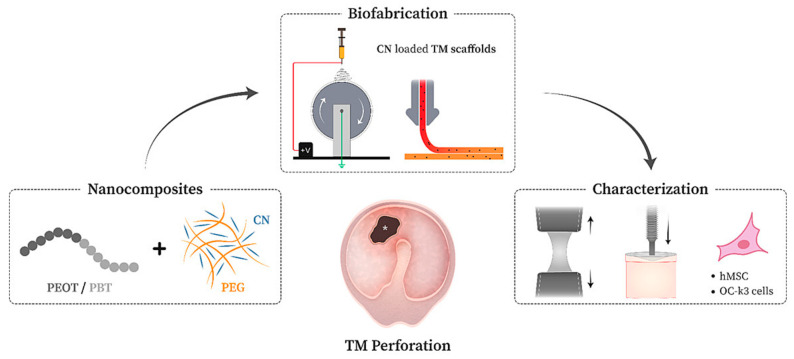
Manufacturing of chitosan-based fibers by electrospinning and investigation of their physical properties and biocompatibility on cell models. Reprinted from Ref. [27].

More complex methods were used to produce films and scaffolds based on chitin, polyethylene glycol (PEG), poly (ethylene oxide terephthalate) and poly (butylene terephthalate) (Figure 2). It is noteworthy that the addition of chitin and PEG to the composite significantly increased mechanical strength and reduced the diameter of the fibers during the manufacture of scaffolds by electrospinning. In addition, films containing chitosan had low toxicity, which was tested on OC-k3 cells (inner ear of mice) and human mesenchymal stromal cells. Also, due to their large surface area, electrospun fibers proved to be a more suitable substrate for human mesenchymal stromal cells than films: cell survival on fibers was 153%, while on films it was less than 60%. However, due to the inclusion of synthetic polymers, the material’s biodegradability is low: about 8% over the course of a year. Low biodegradability is a disadvantage of such composites of natural and synthetic polymers [27].

### 2.3. Hyaluronic Acid

Hyaluronic acid (HA) is a natural glycosaminoglycan that is widely found in the bodies of many animals, including humans. It is found in the skin, joints, eyes and other organs. HA is widely used in tissue surgery and implantology because it is biocompatible and biodegradable, and can retain a significant amount of water, which ensures the hydration of the tissues and cells proliferation around the implant [62].

One of the first studies in this field was conducted by Sten Hellstrom and Claude Laurent. The researchers studied the effect of HA in different concentrations and molecular weights on the healing rate of TM perforations in comparison with PBS and a control group without the treatment. It turned out that the application of a simple HA solution on TM significantly accelerated the healing of it. However, the molecular weight of HA did not have a significant effect on the healing rate. The concentration of HA in the solution was more important: at the concentration of 1.9%, the healing occurred faster than at 1% [29].

Also a work about the effect of an HA solution on TM healing was published. They compared the effectiveness of regeneration when using a 1% HA solution and epidermal growth factor (EGF). The gelfoam particle was soaked in 1% HA solution and then, if necessary, in 400 µg/mL EGF and then applied to the TM perforation. Both substances accelerate TM regeneration in the perforated ear. Interestingly, in the second ear with the perforation, which was left for the control, the TM recovered 2 days faster in the EGF group than in the HA group. EGF is probably able to spread slightly throughout the body after being absorbed into the mucous membrane [30].

**Figure 3:**
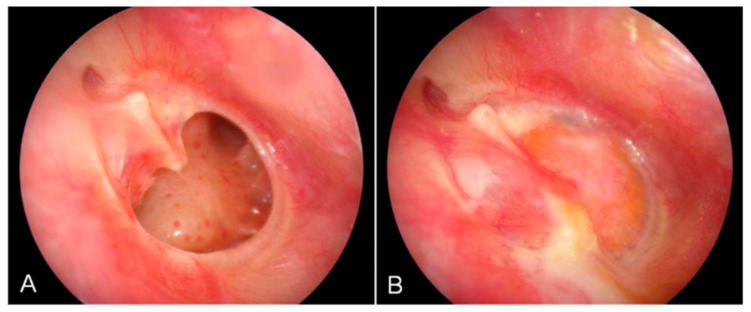
Initial perforation of the tympanic membrane (**A**) and condition of the tympanic membrane 4 months after surgery using hyaluronic acid fat graft myringoplasty. (**B**) The perforation is completely closed and neovascularisation can be identified. Reprinted with permission from Ref. [63] Copyright 2011 Wiley.

Implants based on modified HA or HA and combinations with other materials are also being investigated. It was reported that the combination of HA and the fat graft myringoplasty (FGM) method (i.e., harvesting autologous adipose tissue, usually from the earlobe, as the basis for the implant) promotes effective regeneration of the TM in patients (Figure 3). A fat graft was placed into the TM perforation to close it, and the external auditory canal side was closed with an HA epifilm. However, compared to the use of temporalis fascia or the tragal peri-chondrium (underlay and overlay technique) to close the perforation, the combination of HA and FGM did not show a statistically significant improvement. Nevertheless, the operation with HA plus FGM was completed in four times less time than with other autologous materials, and patients did not need to be hospitalized after the operation, which saves resources and time for patients and doctors [63].

Similar techniques were then tested on pediatric patients (aged from 4 to 16 years). The researchers confirmed their previous results: the combination of HA and FGM had the same therapeutic efficacy as the underlay and overlay techniques, but the operation was performed under local anesthesia rather than general anesthesia. In addition, the postoperative follow-up period for patients was several months shorter. These factors are particularly important in pediatric surgery [31].

Some studies are also investigating the effectiveness of existing surgical materials for TM regeneration. For example, it was reported that in rat models, Epifilm^®^ (HA ether) and Vivosorb^®^ (poly (DL-lactide-ε-caprolactone)) promoted effective TM regeneration within 14 days. An HA ether is a chemically modified form of hyaluronic acid, where the hydroxyl groups on the HA backbone have been modified to form ether linkages by reaction with alkyl groups or other substituents. This modification can change the physicochemical properties such as water solubility, viscosity, stability against enzymatic degradation. Poly (DL-lactide-ε-caprolactone) is a biodegradable copolymer obtained by copolymerization of DL-lactide and ε-caprolactone. It is an amorphous, soft, and elastic polyester copolymer that is used in many biomedical spheres. Commercial films were simply used to cover the TM perforation without any additional fixing or supporting materials. The effectiveness of perforation closure in the natural regeneration and Vivosorb^®^ groups was 85.7% (6/7), while in the Epifilm^®^ group it was 100% (7/7). Epifilm^®^ promotes less neovascularisation and fibrosis than natural regeneration or Vivosorb^®^ [32].

On the other hand, the effectiveness of Epifilm^®^ in 20 patients with TM perforation was investigated, but the film had no effect on the first 5 patients. Films were used to cover the TM perforation also without any fixing. Six weeks after the surgery, the film biodegraded, but the size of the perforation did not change, so the research was stopped [33]. Additional research is still needed to transfer the success from animal models to patients.

A study suggests that HA ether may not always provide optimal conditions. Rats with perforations were either treated with a 1% HA solution daily or covered with MeroGel (surgical tampons made of esterified HA). After 7 days of the experiment, TM control and histological analysis were performed. Vascular endothelial growth factor, fibroblast growth factor, collagen and lymphocytes were observed in both groups. However, in the MeroGel group, the perforation healed in 11/12 rats (91.7%) compared to 12/12 rats (100%) in the HA group. Although this result is not statistically significant [34].

Seprafilm^®^, which contains HA and carboxymethylcellulose, showed a significantly better effect. The material was used without any additions, as in the above-mentioned papers. In the study on rats, the average healing time for perforation in the film group was 7.8 days, while in the natural regeneration group it was 14.9 days. Unfortunately, the authors do not report the area of the perforation created, but the needle diameter mentioned (1.3 mm) suggests that the perforation area was about 50% of the TM area [35].

### 2.4. Collagen

Collagen is a connective tissue protein that participates in the formation of many structures and organs, as well as in the regeneration process. Collagen is plastic, biocompatible, and biodegradable in the human body. Collagen is widely used in tissue engineering as a basis for implants and a structuring component [64].

Collagen combines well with other materials from the human body, even when extracted from other animals. For example, scaffolds were developed on a 3D printer from pig collagen with the inclusion of human umbilical cord serum. Such structures proved to be very effective in regenerating TM in guinea pigs [36]. In another study, membranes were obtained from bovine collagen by electrophoretic deposition. Implants in an experiment on chinchillas contributed to the complete restoration of the TM after the perforation and the return of hearing to normal [37]. Collagen isolated from duck feet also accelerated TM regeneration in rats. At the same time, the material degraded almost completely within 14 days of the experiment, and the collagen patch did not need to be removed, unlike the paper patch [65].

Also it was found out with cell viability and proliferation tests that collagen promote the healing of perforations of tympanic membrane [38]. Thus, the main advantages of collagen are its significant biocompatibility, biodegradability and plasticity, which are especially important for hearing restoration.

### 2.5. Bacterial Cellulose

Bacterial cellulose (BC) is a polymer produced by certain types of bacteria. It consists of microscopic fibers that form a three-dimensional network and has a number of unique properties, such as strength and absorbency. In medicine, it is used as a basis for scaffolds and wound dressings [66].

**Figure 4:**
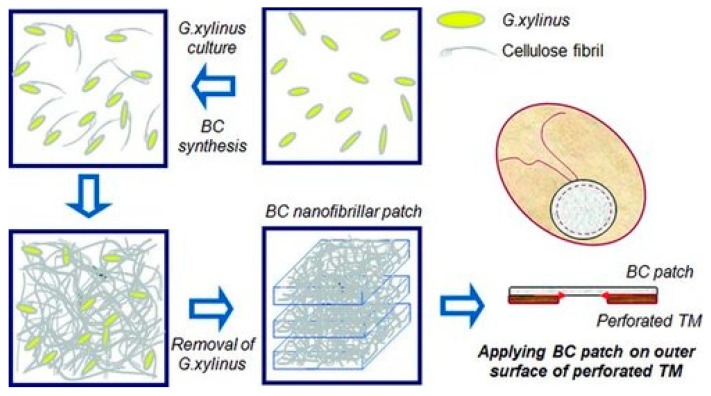
In vitro and in vivo studies demonstrate that the bacterial cellulose nanofibrillar patch promotes the TM healing speed and rate as well as recovering the function of the TM. Reprinted with permission from Ref. [39] Copyright 2013 Wiley.

One of the most known works in this field is an article by Jangho Kim et al. Gluconacetobacter xylinus bacteria synthesized cellulose over a period of 14 days, after which the polymer was collected, purified and sterilized (Figure 4). In vivo, BC-based patches had no significant effect on TM during the first 5 days, but significantly improved regeneration thereafter. Compared to TM that healed naturally, TM healed with BC was closer to the original TM in terms of structure and composition. This study laid the foundation for further work and showed that BC can be a real alternative to other polymers in TM restoration [39].

At the same time, the effectiveness of BC films in comparison with classical methods (TM restoration with autologous temporal fascia and fat graft) was investigated. Each of the three experimental groups consisted of 40 people, of whom 20 had minor perforations and 20 had moderate perforations. In the BC film group, 37 people recovered, compared to 25 in the fat graft group and 30 in the fascia group. The operation time was 15, 35 and 65 min, respectively. Thus, BC-based films are not only better suited for TM regeneration, but also easier for doctors to use [40].

Other polymeric films were also studied. For example, the Bionext^®^ film has been developed for wound closure and skin regeneration. Bionext^®^ is bacterial cellulose film for wound treatment, used as a temporary skin substitute for burns, injuries, chronic and diabetic ulcers. It was investigated how this film affects TM healing in 24 patients. Immediately after applying the film, the patients’ well-being improved and their hearing sensitivity increased. A month later, the patients were invited for a follow-up examination, and most of those (83%) had fully recovered, while the rest recovered after another 2 months. BC showed excellent results, however more research is needed in clinical trials to monitor the recovery of all patients [41].

Another BC-based graft from Polisa™, biopolymer membrane obtained by polymer synthesis from sugarcane molasses, has successfully completed clinical trials on 40 patients. All patients were divided into two groups: those with a BC-based graft and those using autologous temporal fascia for TM restoration. In the BC group, regeneration was faster and more complete, the operation time did not exceed 15 min, and the cost of therapy per patient was 13 times lower than in the fascia group. However, the authors do not provide data on the composition and production process of the transplant. Therefore, additional research is needed to determine the components of successful BC application [42].

It can be concluded that BC is one of the most promising materials for TM regeneration, as it has an extremely positive effect on regeneration. In addition, BC production can potentially be scaled up, thereby making the method even more cost-effective.

## 3. Other Polymers

Many other natural polymers are also used for TM regeneration, but they are not as frequently encountered in research as the polymers mentioned above. In this section of the review, we will discuss the use of less popular polymers.

### 3.1. Poly(l-lactic acid) and Poly(lactic-co-glycolic acid)

Poly(l-lactic acid) (PLA) and poly(lactic-co-glycolic acid) (PLGA) are both biodegradable and biocompatible polymers used in various biomedical applications. PLA is a homopolymer of lactic acid, while PLGA is a copolymer of lactic acid and glycolic acid. The polymers are often used together as a base solution for electrospinning.

**Figure 5:**
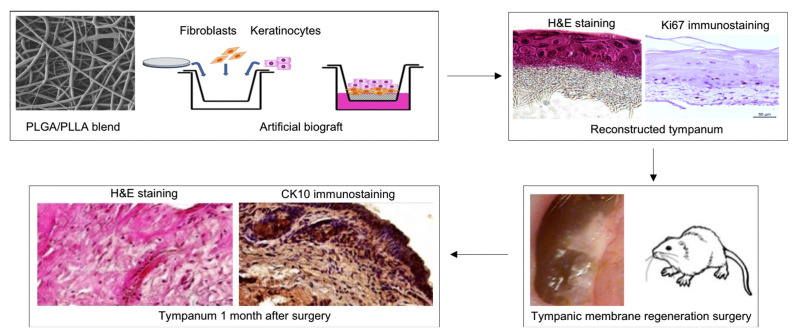
The process of creating and researching in vivo artificial grafts based on PLGA/PLLA with the inclusion of fibroblasts and keratinocytes for TM regeneration. Reprinted with permission from Ref. [43]. Copyright 2017 Elsevier.

Electrospun scaffolds were used for TM regeneration. Scaffolds with immersed fibroblasts and fibroblasts with keratinocytes showed greater efficacy than the scaffolds alone (Figure 5). In rats with simple polymer matrices, rough traces of regeneration were observed, as well as side effects: cholesteatomas and granulomas. Scaffolds are necessary for such artificial tissues, as the cells themselves are too vulnerable and do not hold their shape during transplantation [43].

In addition to being used as a base for implants with cells, PLA is also used to create implants that are similar in acoustic properties to native TM. The matrix is also formed by electrospinning. Thus, in a series of experiments with different polymers with the addition of graphene oxide, the PLA-based implant showed the best acoustic properties. Further research is needed to investigate the biocompatibility and safety of this matrix [67].

### 3.2. Latex

Natural latex is a substance obtained from the Brazilian rubber tree (*Hevea brasiliensis*). Latex is a complex emulsion consisting of various components, including proteins and resins. Due to its elasticity and strength, latex is widely used in regenerative medicine for the restoration of bones in joints, cartilage, and also in dentistry [68].

Latex has potential for use in otosurgery, as some biocompatibility studies have already been conducted on cell and animal models [69,70]. Latex has also been tried for TM regeneration. In one work authors compared latex film with Sylastic^®^ silicone film, a type of silicone implant material used in otorhinolaryngology surgery for a variety of purposes, when performing classic underlay myringoplasty technique surgery. In the group of patients treated with latex, 66% of people had complete TM healing, compared to 55% in the case without lining and 57% in the case of Sylastic^®^. Also, vascularisation was more active in patients treated with latex, which the authors attributed to the presence of growth factors in *Hevea brasiliensis* that could affect human tissue. Additional research remains to be performed in this area, as the percentage of healed TM is still too low, and it is known that latex can cause allergic reactions as it is a foreign polymer [44].

### 3.3. Gelatin

Gelatin is a product of partial hydrolysis of collagen. It is mainly used in cooking but also finds application in pharmaceuticals and cosmetology [71]. Gelfoam^®^ (Pharmacia & Upjohn Company LLC, Kalamazoo, USA) foam has been developed on the basis of gelatin and is widely used for local control of bleeding during surgery. In fact, in many articles mentioned in the review, Gelfoam^®^ is used in surgical invasions.

For TM regeneration, gelfoams without additional substances were tried and they showed excellent results in terms of both the degree of TM healing and the time of regeneration [45]. That is, such matrices themselves create favorable conditions for cell proliferation. However, such studies using only Gelfoam are very limited. Gelatin matrices are usually used as an auxiliary material or loaded with active substances. For example, an experiment with guinea pigs showed that when fibroblast growth factor was included in the gelatin matrix, the TM in all animals in the group recovered within 8 days after laser perforation. In addition, the structure of the restored TM is identical to native TM and includes epithelial, fibrous and mucous layers and blood vessels [46]. It is also important to note that gelatin is used to create fibers by electrospinning, using synthetic and natural cross-linkers. For example, modification of gelatin with methacrylate and subsequent cross-linking with tannic acid has a positive effect on the mechanical properties of the fibers, as the acid promotes the formation of numerous hydrogen bonds between hydrogen in hydroxyl and amino groups and oxygen. The strength of scaffolds, in turn, is important for imitating the acoustic properties of natural TM. In an experiment on TM regeneration in vivo, such scaffolds performed better than simple gelfoam, probably because the cross-linked structure is more suitable for cell proliferation than only gelatin itself [72]. It is also possible to crosslink the gelatin matrix using genipin. Researchers have investigated electrospun gelatin structures crosslinked with genipin as potential scaffolding materials for tympanic membrane regeneration, highlighting their non-toxic nature, favorable mechanical characteristics, biocompatibility and durability [47].

Gelatin matrices are an excellent basis for implants; they are widely used not only in surgical operations but also in combination with other polymers, as we will see later.

### 3.4. Polycaprolactone

Polycaprolactone (PCL) is a biodegradable and biocompatible polyester synthesized from ε-caprolactone. PCL, like PLA and PLGA, is widely used as a polymer for electrospinning, for the regeneration of vessels, bones and skin [73].

**Figure 6:**
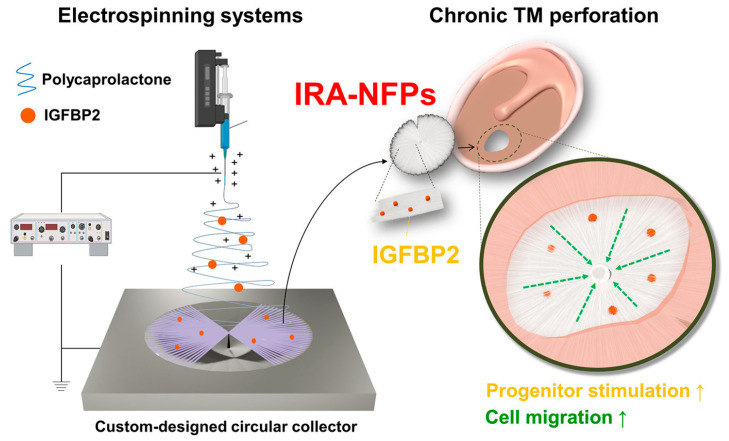
Electrospun radially aligned nanofibrous patches loaded with insulin-like growth factor binding protein 2 (IGFBP2) significantly improved TM regeneration in animal models. Reprinted from Ref. [28].

Electrospun fibers can be loaded with active substances and cell cultures, and the porosity of scaffolds promotes cell proliferation and implant ‘ingrowth’ into tissue. Therefore, PCL scaffolds have also been tried for TM regeneration. For example, in one study, electrospun scaffolds with epidermal growth factor significantly improved TM healing in vivo [48] (Figure 6). In a subsequent study, authors immersed insulin-like growth factor binding protein 2 (IGFBP2) in PCL fibers. The scaffolds helped speed up the healing of TM perforations, even though the maximum perforation area in the groups did not go over 20% [49].

It is important to note that in both studies, the authors used radially aligned electrospun fibers, meaning that in the TM they created, the fibers were arranged along the radius. Even without growth factor or IGFBP2, such matrices promoted regeneration, probably by directing cell proliferation along the fibers. In other words, the shape of the matrix can significantly affect the rate of regeneration.

### 3.5. Alginates

Alginates are salts of alginic acid, a natural polysaccharide obtained from brown seaweed. They are widely used in various fields, including the food industry, medicine, cosmetics, and dentistry. Alginates have unique properties, such as the ability to form gels, bind water, thicken and stabilize mixtures [74]. Alginate hydrogels are considered a promising material for creating implants, as they are highly compatible with tissues, easy to form into a gel, and inexpensive to produce [75].

The ability of alginates to retain water is used in transplantology to create patches and fibers that would maintain optimal conditions for TM regeneration. Thus, alginate-based patches were developed, which in an experiment with chinchillas showed significantly greater effectiveness than paper patches. The authors used calcium ions as a cross-linker for polymer molecules and found that a 4% ion concentration slightly impaired TM regeneration compared to a 2% concentration. However, patches with a 4% ion concentration were more durable and resistant to damage. Therefore, studies on both animals and patients are needed to determine the necessary crosslinker concentrations. This applies not only to ionic crosslinkers but also to other substances [50].

In addition to using alginate on its own, it is also combined with synthetic polymers. For example, electrospun fibers based on polyvinyl alcohol (PVA) and alginate (50:50) provided the best conditions for NIH/3T3 cells (mouse embryonic fibroblasts) in vitro. And in an experiment with rats, the PVA/alginate + Wharton’s Jelly (mucoid connective tissue that surrounds the umbilical cord vessels) composite showed excellent results in regeneration and hearing restoration. But in the case of such a non-standard donor material as Wharton’s Jelly, its source must be considered in advance when scaling up the method [51].

## 4. Composites Based on Several Polymers

As we have seen in previous sections, natural polymers have useful characteristics. To give implants optimal properties, composites are being developed that combine not only several natural polymers, but also natural and synthetic polymers. This makes it possible to achieve several useful properties at once.

As in the case of single polymers, different methods are used to manufacture TM from composites. One of the simplest is the Petri dish pouring method. This is how films based on silk fibroin and gelatin are obtained. The two polymers are cross-linked with genipin, and such films have shown excellent results in terms of both TM regeneration rate and biocompatibility [52]. The pouring method is also used to produce patches with a chitosan-collagen net structure, with glycerol used as a plasticizer [76]. In both cases, the polymers have the same charge or no charge at all, but their interaction is ensured by a cross-linking agent (genipin) or intertwining chains (chitosan and collagen). Another possible combination of polymers and cross-linking agent: polyvinyl alcohol and gelatin are cross-linked with maleic anhydride to form a gel with a stable structure. The gel showed excellent mechanical properties, but in an in vitro cytotoxicity test, the survival rate of L929 cells (mouse fibroblasts) decreased to almost 80%. This is probably due to the large amount of maleic anhydride compared to PVA (1:1). In the future, the authors should work on the biocompatibility of the gel and test it on animal models [77].

Among non-standard methods of polymer modification, there is a gel based on HA and chitosan, which is fixed with a CO_2_ laser. The gel was studied in comparison with EpiDisc (etherified HA) on chinchillas. Although more animals recovered in the gel group, the gel had to be reapplied to some animals, resulting in middle ear infection in 6/26 chinchillas. In the EpiDisc group, the TM closed in only half of the animals, but the TM thickness was closer to the initial value than in the gel group. So far, photocurable gel has shown insignificant advantages in therapy, so further research on its use is needed [53].

**Figure 7:**
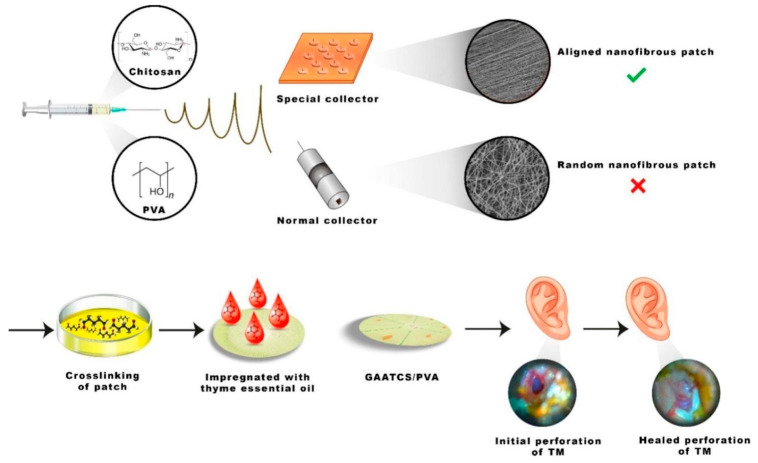
Aligned nanofibers based on chitosan/PVA composite loaded with thyme essential oil have a significant antibacterial effect and good biocompatibility. Reprinted with permission from Ref. [54] Copyright 2023 Elsevier.

Electrospinning is often used to create scaffolds for TM regeneration. This method is also used to manufacture composites, often using cross-linkers to improve mechanical properties and increase material stability. The possibility of creating aligned patch using electrospinning rather than 3D printing was demonstrated. The printed fibers were cross-linked with glutaraldehyde and loaded with thyme essential oil to obtain antibacterial properties (Figure 7). The fibers showed a significant zone of inhibition of *Pseudomonas aeruginosa* (25 mm) and *Escherichia coli* (25 mm) in the disk diffusion method. However, in the cytotoxicity test, the survival rate of mesenchymal stem cells decreased to 81%. The authors conducted preliminary successful trials on six patients with TM perforation, but number of patients needs to be increased to obtain reliable data [54].

Fibers created by electrospinning can also be used as a component of implants. For example, PCL and gelatin fibers were used to create a composite with alginate hydrogel. The resulting sandwich structure promoted rapid healing of the TM, and the presence of exosomes in the alginate hydrogel promoted natural regeneration. Such an original implant has great potential, but it takes a significant amount of time to produce [55]. Another example of the use of human tissue in a composite is presented in the work of Hyeongjin Lee et al. The researchers developed an implant based on PCL and silk fibroin and included human umbilical cord serum as the active ingredient. The composite showed excellent results in in vivo and in vitro experiments compared to a paper patch. However, it would also be interesting to see the results of a comparison of fibers with and without human umbilical cord serum to assess the effect of cell factors and active substances [78].

It is also possible to combine collagen and poly (ε-caprolactone) structures to achieve properties similar to those of natural TM. Human mesenchymal stromal cells (hMSCs) were cultured on star-branched poly (ε-caprolactone) (*PCL) scaffolds. Under dynamic conditions, hMSCs showed fibroblast marker overexpression and produced collagens I, II, III, and IV. This approach demonstrates that *PCL scaffolds with appropriate mechanical properties can guide hMSCs toward TM-like fibroblast collagen production [79].

Another advantage of electrospinning is the ability to encapsulate a large amount of active substances in the fibers and then control their release by changing the morphology of the fibers. PLGA copolymers were first investigated for its mechanical properties [56]. Then one of the studies was focused on the use of poly (ethylene oxide terephthalate)/poly (butylene terephthalate) (PEOT/PBT) and poly (d,l-lactide-co-glycolide) (PLGA) to create fibers and incorporate the antibiotic ciprofloxacin into them. The solvents used were trichloromethane and absolute ethanol for PEOT/PBT and water with Span 80^®^ for PLGA. Different polymer ratios were used to create different drug release profiles. It is also interesting that the addition of the antibiotic increased the mechanical strength of the fibers. The authors have successfully tested the fibers in vitro for restoration of TM. Experiments on animals are planned in future [80].

Another area of the application for composites is 3D printing of TM. This technology allows grafts to be produced with specified shape and structure parameters, which provides control over the properties of the transplant. Well-known polymers such as gelatin are used to create the basis for such structures. By combining gelatin and methacrylate-modified gelatin, it is possible to obtain layered structures and then wash out the gelatin layers. In this way, the graft for TM restoration was obtained. The graft showed excellent results in an in vivo toxicity test, and the authors conducted an experiment on animal models. In groups with perforation without the treatment, with a graft, and with a graft loaded with EGF, the following results were observed for TM restoration within 14 days: 1/4, 3/4, and 6/6, respectively. However, the small number of animals used in the experiment requires further research to confirm the effectiveness of such a graft [57]. The combination of the scaffold and EGF can significantly improve the healing rate of the eardrum. The factor mainly affects the epithelial cell layer, promotes keratinocyte migration and capillary regeneration, and increases collagen formation in the regenerating tissues [81].

Ideas inspired by nature are also used in the development of transplants. For example, one graft was printed in two approaches: first, a silk scaffold was printed, and then the surrounding area was filled with a fibrin-collagen composite hydrogel matrix [82]. Unfortunately, the authors only investigated the mechanical properties of the transplant. Such a structure may provide not only mechanical strength but also guide cell proliferation during regeneration, as we saw in the case of PCL scaffolds [49].

The most complex composites can contain more than three polymers. For example, in one study, scaffolds with different numbers of fibers (8 or 16) were made from PDMS, PLA and PCL and filled with a fibrin and collagen hydrogel. The grafts were then tested using laser and holographic methods. The resulting 3D structures showed more regular and stable oscillatory characteristics in response to sound compared to human fascia. In addition, their mechanical strength and load resistance were significantly higher [83].

Composites are also being developed that can release not only growth factors but also their receptor genes. Such structures can increase adhesion, proliferation, migration ability and viability of tympanic membrane cells. The matrix for factors and genes can be a polyethyleneimine (PEI)/chitosan composite, which combines the properties of natural and artificial substances [84].

## 5. Conclusions

The development of implants for TM regeneration is an important area in modern medicine, which involves many parameters. Natural polymers, which are already widely used in tissue surgery, and new polymers, which have rarely been used before, are used to create materials. At the same time, in each individual case, there are many parameters that can be varied to obtain the necessary properties of the implant: polymer molecular weight, polymer concentration, presence and concentration of cross-linkers, the manufacturing method, and others.

Many polymers demonstrate very perspective properties and have potential for use in otologic surgery, but several issues in this area still need to be resolved. For example, the biodegradation of polymers must be controlled, as rapid degradation prevents complete regeneration of the TM, while degradation time that is too long will cause discomfort to the patient. In addition, work needs to be performed on biocompatibility so that the stem cells placed in the implant remain alive for a long time. Another significant problem is the variability in the biological composition between batches of natural materials, which leads to inconsistent treatment results. Thus, natural polymers require modification or combination with synthetic materials to meet clinical requirements [85].

Research is already underway in a very promising area: the inclusion of cell cultures in grafts. It has been found that mesenchymal stem cells (MSCs) can differentiate into epithelial cells that produce many growth factors and cytokines that stimulate wound healing. These assumptions have been confirmed in in vivo experiments on animal models of rats and pigs [86]. In addition to MSCs, epidermal cells derived from the tympanic membrane with stem cell-like characteristics may also play a role in the regeneration of perforations [87].

It seems possible that in the future there will be two main directions for TM restoration: emergency restoration on site and deep regeneration. In the first case, the operation can be performed without prior preparation using simple films made of natural polymers to close small perforations. In the second case, complex implants with a special structure are used, created by electrospinning or 3D printing with the inclusion of stem cells and a set of growth factors, such as insulin and EGF [88]. In each of the two cases, materials with specific properties are required, which have yet to be developed and tested in clinical trials.

## Figures and Tables

**Table 1:** Main characteristics of the studied structures based on natural polymers.

	Implant Composition	Polymer Molecular Weight	Polymer Graft form/Active Inclusions and Substances	Subject of Experiments		% of Subjects with Restored TM	Reference
				Cell Lines	Animals and Human		
1	Silk fibroin	naturally derived	casted polymer patch/-	-	rats	93%	[19]
2	Silk fibroin	naturally derived	casted polymer patch/-	L929 cells	rats	-	[20]
3	Silk fibroin	naturally derived	casted polymer patch/-	-	rats	100%	[21]
4	Silk fibroin	naturally derived	casted polymer patch/-	-	guinea pigs	100%	[22]
5	Silk fibroin Tympasil^®^ patch	-	ready film/-	-	human	70%	[23]
6	Silk fibroin	naturally derived	casted polymer patch/-	human TM cells	-	-	[24]
7	Chitosan	100–200 kDa	casted polymer patch/-	human TM cells	rats	100%	[25]
8	Chitosan	200 kDa	freezing and lyophilizing of polymer solution/-	human TM cells	rats	100%	[26]
9	PEOT/PBT/(Chitin/PEG)	Chitin: naturally derived PEOT/PBT: 93 kDa PEG: 4000 g/mol	compression molding/-	human mesenchymal stromal cells OC-k3 HaCaT PC12	-	-	[27]
10	Chitosan	200 kDa	casted polymer patch/IGFBP2	human TM cells	rats	44%	[28]
11	HA	4 or 6 MDa	polymer solution/-	-	rats	100%	[29]
12	HA	-	polymer solution/-	-	rats	100%	[30]
13	HA epidisc	-	fat graft myringoplasty	-	human	93%	[30]
14	HA epidisc	-	fat graft myringoplasty	-	human (children)	87%	[31]
15	HA ester Epifilm^®^	-	ready epidisc/-	-	rats	100%	[32]
16	HA ester Epifilm^®^	-	ready epidisc/-	-	human	0%	[33]
17	esterified HA MeroGel^®^	-	ready surgical tampons/-	-	rats	92%	[34]
18	Seprafilm^®^	-	ready film/-	-	rats	100%	[35]
19	Collagen	-	3D printing/umbilical cord serum	NHDFs	guinea pigs	100%	[36]
20	Collagen	naturally derived	electrophoretic deposition/-	-	chinchillas	100%	[37]
21	Collagen	naturally derived	lyophilisation of polymer solution/-	-	rats	100%	[38]
22	Bacterial cellulose	naturally derived	naturally formed/-	rat TM cells	rats	100%	[39]
23	Bacterial cellulose	naturally derived	naturally formed/myringoplasty	-	human	92.5%	[40]
24	Bionext^®^	-	ready film/-	-	human	100% of examined patients	[41]
25	BC graft from Polisa™	-	ready film/-	-	human	90%	[42]
26	PLLA and PLGA	PLLA: 216,000 g/mol PLGA: 110,000 g/mol	electrospinning/fibroblasts and keratinocytes	human fibroblasts	rats	100%	[43]
27	Latex	naturally derived	-/underlay myringoplasty	-	human	67%	[44]
28	Gelatin gelfoam	-	-/-	-	human	97%	[45]
29	Gelatin	-	chemically cross-linked hydrogel/basic fibroblast growth factor (bFGF)	-	guinea pigs	100%	[46]
30	Gelatin and methacrylic acid	-	electrospinning/-	HEI-OC1 cells	guinea pigs	100%	[47]
31	PCL	80 kDa	electrospinning/epidermal growth factor (EGF)	rat TM cells	rats	100%	[48]
32	PCL	80 kDa	electrospinning/IGFBP2	rat TM cells	rats	100%	[49]
33	Calcium alginate	-	injection molding technology/-	-	chinchillas	71%	[50]
34	Alginate sulfate and PVA	Alginate sulfate: 140 kDa PVA: 85,000–124,000 g/mol	electrospinning/Wharton’s Jelly	NIH 3T3	rats	100%	[51]
35	Silk fibroin and gelatin	naturally derived	casted polymer patch/-	human dermal fibroblast (HDF)	rats	100%	[52]
36	HA and chitosan	-	photocurable gel/-	-	chinchillas	100%	[53]
37	Chitosan and PVA	Chitosan: 600 g/mol PVA: 88–98 kDa	electrospinning/thyme essential oil	*Staphylococcus aureus*, *Pseudomonas aeruginosa*, *Escherichia coli* and Mesenchymal stem cells	human	100%	[54]
38	PCL, gelatin and sodium alginate	PCL: 80,000 g/mol sodium alginate: 200–300 kDa	composite structure/exosomes from human adipose-derived mesenchymal stem cells	NIH/3 T3	rats	100%	[55]
39	PCL and silk fibroin	PCL: 80,000 g/mol silk fibroin: naturally derived	composite structure/umbilical cord serum	human dermal fibroblasts	guinea pigs	100%	[56]
40	Gelatin and gelatin modified with methacrylic anhydride	-	3D printing/EGF	NIH/3T3	chinchillas	100%	[57]

## Data Availability

No new data were created or analyzed in this study. Data sharing is not applicable to this article.
